# Community-based geographical distribution of *Mycobacterium ulcerans* VNTR-genotypes from the environment and humans in the Nyong valley, Cameroon

**DOI:** 10.1186/s41182-021-00330-2

**Published:** 2021-05-21

**Authors:** Francis Zeukeng, Anthony Ablordey, Solange E. Kakou-Ngazoa, Stephen Mbigha Ghogomu, David Ngolo Coulibaly, Marie Thrse Ngo Nsoga, Wilfred Fon Mbacham, Jude Daiga Bigoga, Rousseau Djouaka

**Affiliations:** 1 The Biotechnology Centre (BTC), University of Yaound I, P.O. Box, 17673 Yaound, Cameroon; 2 Department of Biochemistry and Molecular Biology, Faculty of Science, University of Buea, P.O. Box., 63, Buea, Cameroon; 3 Department of Bacteriology, Noguchi Memorial Institute for Medical Research, University of Ghana, P.O. Box., 581, Legon, Accra, Ghana; 4 Department of Technics and Technology, Platform of Molecular Biology, Pasteur Institute Abidjan, P.O. Box., 490, Abidjan 01, Abidjan, Cte dIvoire; 5 Akonolinga District Hospital, P.O. Box., 18, Akonolinga, Cameroon; 6 The AgroEcoHealth Platform, International Institute of Tropical Agriculture (IITA), 08 P.O. Box. 0932, Tri-Postal Cotonou, Cotonou, Bnin

**Keywords:** *Mycobacterium ulcerans* infection, VNTR-profiling, Locus repeat, Environmental samples

## Abstract

**Background:**

Genotyping is a powerful tool for investigating outbreaks of infectious diseases and it can provide useful information such as identifying the source and route of transmission, and circulating strains involved in the outbreak. Genotyping techniques based on variable number of tandem repeats (VNTR) are instrumental in detecting heterogeneity in *Mycobacterium ulcerans* (MU) and also for discriminating MU from other mycobacteria species. Here, we describe and map the distribution of MU genotypes in Buruli ulcer (BU) endemic communities of the Nyong valley in Cameroon. We also tested the hypothesis of whether the suspected animal reservoirs of BU that share the human microhabitat are shedding contaminated fecal matters and saliva into their surrounding environments.

**Methods:**

Environmental samples from suspected MU-risk factors and lesion swabs from human patients were sampled in BU-endemic communities and tested for the presence of MU by qPCR targeting three independent sequences (IS*2404*, IS*2606*, KR-B). Positive samples to MU were further genotyped by VNTR with confirmation by sequencing of four loci (MIRU1, Locus 6, ST1, Locus 19).

**Results:**

MU was detected in environmental samples including water bodies (23%), biofilms (14%), detritus (10%), and in human patients (73%). MU genotypes D, W, and C were found both in environmental and human samples. The micro geo-distribution of MU genotypes from communities showed that genotype D is found both in environmental and human samples, while genotypes W and C are specific to environmental samples and human lesions, respectively. No obvious focal grouping of MU genotypes was observed at the community scale. An additional survey in the human microhabitat suggests that domestic and wild animals do not shed MU in their saliva and feces in sampled communities.

**Conclusions:**

VNTR typing uncovered different MU genotypes circulating in the endemic communities of the Akonolinga district. A MU environmental genotype was found in patients, yet the mechanism of contamination remains to be investigated; and recovering MU in culture from the environment remains key priority to enable a better understanding of the mode of transmission of BU. We also conclude that excretions from suspected animals are unlikely to be major sources of MU in the Nyong Valley in Cameroon.

**Supplementary Information:**

The online version contains supplementary material available at 10.1186/s41182-021-00330-2.

## Background

Despite progress, particularly in understanding the risk factors for Buruli ulcer (BU), the isolation of *Mycobacterium ulcerans* (MU) from patients and the environment around them is key to a useful understanding of how BU spreads. Different hypotheses regarding the niche environments and transmission pathways of MU have been proposed. For many of these, the predilected environment for this pathogen is aquatic, and MU is involved in different transmission pathways, which could be biological through insect bites [1,3] or mechanical through skin trauma and contact with MU-contaminated environments [4, 5]. To design a significant public health intervention, determining the transmission pathways remains a key research priority.

A strong association exists between BU outbreaks and close proximity to stagnant water bodies [6,10]. Numerous investigations of the environment have attempted to identify the pathogens source with so far, only limited success. Although environmental studies on the sources of MU have detected signature sequences in the host of samples including water bodies [11, 12], detritus and plant biofilms [12, 13], aquatic insects and animals [13, 14], mosquitoes [15, 16], domesticated animals [1, 17,19], peri-domestic small mammals [12, 20, 21], and wild animals [1, 22,24]; it remains challenging to recover MU in culture from the environment [25].

There is a gap of information on the extent of MU transmission between environmental elements and how humans and animals are colonized or infected in the contaminated environment. SNPs and whole-genome sequence (WGS) comparisons of clinical isolates of MU have enabled mapping the distribution of MU genotypes within local village and district levels [26,29]. All of these analyses invariably identified local MU genotypes that form focal transmission clusters. There was no mixture of genotypes at a locality scale; however, Ablordey et al. [27] showed for the first time the occurrence of multiple clinical MU genotypes at a local scale.

The distribution pattern for environmental MU genotypes has however not been possible as a result of the difficulties in recovering MU in culture from the environment. As high-resolution SNPs and WGS analyses cannot be performed on environmental samples, these methods, therefore, have limited applicability for analyzing potential epidemiological links between patients and environmental sources of MU. On the other hand, variable number of tandem repeats (VNTR) profiles have been directly determined for MU in environmental samples [30, 31] and have been used to track MU isolates in the colonized environment and follow transmission chains to humans [30]. It is also taught that the environment can be contaminated with MU shed from BU lesions or fecal matters of animals like possums [22], agouti (*Thryonomys swinderianus*) [32], and humans [33]. However, further investigations are needed in Africa, especially in endemic areas where BU-like lesions have been found in small mammals and domesticated animals [12, 17, 20, 32].

MU distribution is far broader than the distribution of human disease in high endemic foci. Focal demography and social water contact patterns may play a significant role in transmitting the disease [30]. In a study describing the health impacts of environmental mycobacteria, Primm et al. [34] reported that transmission of environmental mycobacteria is dependent on the overlapping habitats of the pathogen and humans. This hypothesis was tested in BU endemic communities located in the Nyong valley in Cameroon.

In this study, we determined the VNTR genotypes of MU from the environment and humans and mapped their distribution in the BU endemic communities of Akonolinga district. We also assessed whether the suspected animal reservoirs of BU that share the human microhabitat are shedding contaminated fecal matters and saliva into their surrounding environments. This contamination may occur during uncontrolled defecations in the surrounding risk-environment like water bodies, and during bathing and feeding activities.

## Methods

### Study sites

The study is part of field investigations conducted between 2016 and 2017 in the Nyong valley in the Centre region of Cameroon. We conducted field activities in six BU endemic communities in the Akonolinga health district. The communities where investigations were made share various streams from the Nyong river and the districts main BU-risk factor [9]. Various environmental elements were sampled round suspected waterbodies at Akonolinga Centre (3 46 30 N; 12 14 54 E), Yeme-Yeme (3 34 8 N; 12 10 2 E), Nyeck (3 50 34 N; 12 18 32 E), Edjom (3 32 20 N; 11 58 2 E), Endom (3 30 14 N; 12 5 46 E), and Nkolessong (3 52 10 N; 12 19 33 E). These communities constitute the health areas administrative centers, and each has an integrated health center (CSI) or a district medical center (CMA). The attributes of each study site, including the BU-risk factors and prevalence, as well as the selection criteria of sites, have been described previously [17]. The inhabitants of these communities mainly use water from the streams, rivers, and ponds for domestic needs and agricultural activities. Overall, risk factors for BU in the district include wading in swamps and having activities (bathing, cleaning, drinking, and mining) in the Nyong river [9]. These factors may contribute to the spreading of MU within the communities. Hunting also constitutes a major activity in the Nyong valley and specifically in Akonolinga and Ayos, another endemic district for BU. Some large bushes and forests here host different wild mammals (antelope, porcupine, hedgehog, rats, tiger cat, monkey, etc.). It is frequent to find various types of animal feces in the human microenvironment. Since several cases of MU-infected wounds have been described in mammal species across Africa [12, 17, 20, 32], we tested if these animals can harbor MU in their feces and saliva in BU endemic communities.

### Environmental and clinical sampling

We carried out a campaign of environmental investigation of MU from aquatic elements (water bodies, detritus materials, plant biofilms) and terrestrial materials (feces and saliva swabs from wild and domesticated animals). We further overlapped the distribution of each sample in the sampled communities, as well as the MU genotypes found.

#### Water filtrands

**Fig. 1:**
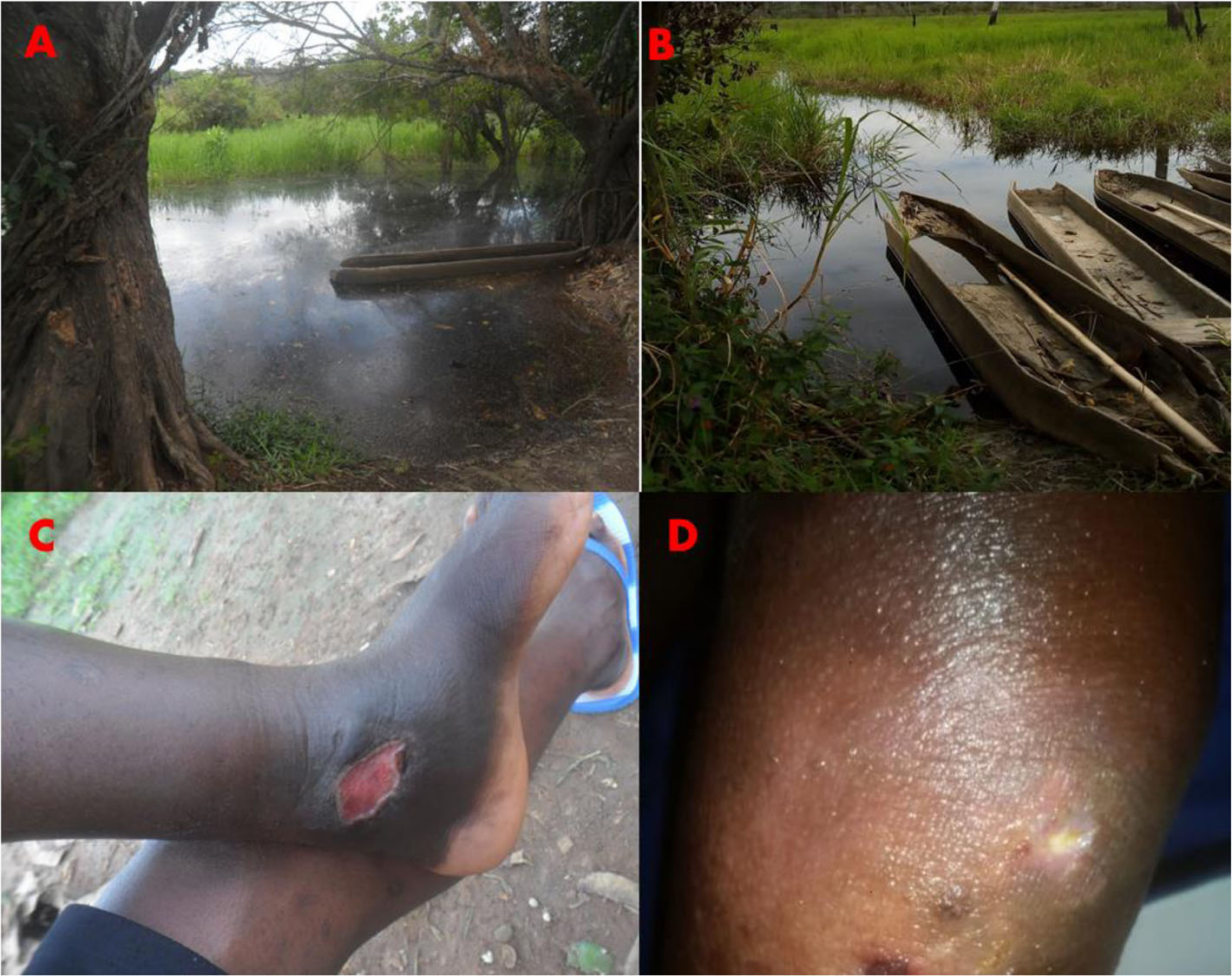
Two water bodies and two suspected BU-like lesions that were sampled at Edjom. **a** Nyong river (Nlong). **b** Fishing activities on the Nyong river. **c** Suspected BU-lesion (a circular ulcer of the left foot at the granulation phase on an articulation. This is a venous ulcer with a dark background and edema of the foot, found in a 27-year-old fisher. **d** Suspected BU-lesion (a plate of the left elbow with the onset of ulceration and a fibrinous background, found in a 14-year-old boy). These patients declared to work in close contact with the Nyong river for fishing and agricultural activities

Water specimens were sampled from suspected stagnant water bodies (streams, rivers, puddles, swamp) using previously described protocols [12, 35]. Investigated water bodies corresponded with suspected niche environments for MU (Fig. 1). All collected water bodies with local naming and test IDs are provided as supporting information to this article (Additional file 1). Briefly, 1000 ml of water was sampled into sterile glass bottles by immersion from the water bodys surface. A sterile glass-spoon was used to collect water samples from puddles around the health units or the houses of local traditional healers specialized in the treatment of chronic wounds. The bottles were washed between sampling sites, autoclaved, and rinsed with double-distilled water before water sampling. A composite sample consisting of different sampling spots of the same water body was constituted, and the bottles were covered with aluminum foil, and transported to the laboratory. Each bottle was then shaken vigorously, and 100 ml or less of water was filtered through a 0.45 m Whatman nitrocellulose filter (Whatman). The filter was removed after the filtration and wrapped entirely in aluminum foil. The process was duplicated, and the samples kept at +4 C until molecular processing.

#### Plant biofilms and detritus materials

Plant biofilms were also collected from stems and leaves of dominant aquatic vegetation in sampled water bodies using a modified protocol adapted from Williamson et al. [30] and Narh et al. [12]. Collected specimens were kept in sterile Ziplock bags, and the biofilms were dislodged by rubbing the bag vigorously several times to make a composite. The composite was kept fresh for further laboratory processing. Detritus materials resulting from the decomposition of organic matters (plants, garbage, animals) were collected by composite within water bodies and from the ground using a sterile glass-spoon. Collected specimens (~200 g) were treated as per the biofilms and kept for further investigations.

#### Fecal materials

Before this study, we did not assume which type of fecal material might have been positive to MU. However, only specimens from suspected animal reservoirs were investigated, including fecal matters from wild mammals (porcupine, hedgehog, rats), domestic animals (canine, ovine, avian, porcine), and peri-domestic small mammals (mice). Sampling of animal specimens was performed in BU endemic communities including houses where BU cases were found in patients, and near BU risks factors like watercourses. A sampling of sites was systematically performed using 10-m intervals along straight line transects and starting from a water source used for domestic or agricultural activities. Fecal materials shed on the soil were identified according to the physical shape and appearance. A composite sample was made before the collection of materials distributed in 1 m diameter according to animal species. All specimens were collected in duplicate round the watery environment, bushes or fields, and the houses. Briefly, the sampling process consisted in locating any fecal material deposited on the soil, followed by direct identification according to the physical shape or appearance. A hunter was contacted for the identification of unknown feces shed by wild animals. A sterile glass-spoon was used to collect 100200 g of feces into clean Ziplock bags, and collected specimens were kept at 20 C until laboratory processing.

#### Saliva swabs from animals

Roaming domesticated animals living in close habitat with human beings were kept in a paddock, as described previously [17]. After the identification of animal species, clinical and anthropological parameters were recorded. Swabs with wooden shafts were used to collect saliva and feces specimens using the modified protocol of the World Health Organization [36]. Saliva and feces swabs were respectively collected in duplicate from the mouth and anus of the animals. Then, collected specimens were placed immediately into sterile tubes and kept in a cooler box. Samples were systematically collected from animals carrying BU-like and unlike lesions and those with no skin/soft tissue lesion.

#### Lesion swabs from suspected BU patients

Clinical swabs from suspected or confirmed BU active lesions were collected simultaneously during field investigations, using the WHO recommended protocol [36] (Fig. 1). Patients not following any antibiotic-based treatment regimen of the WHO guidelines were referred to the nearest health Center or Akonolinga district hospital to manage the wounds properly. Collected samples were confirmed by PCR, and results sent back to the hospital as recommended by the national Buruli ulcer control program. No microscopy and culture were attempted for collected human samples.

### Molecular analyses

#### Extraction of genomic DNA

Overall, 250 mg composite of each solid element (fecal matters, detritus, and plant biofilms) were crushed in sterile mortars with 2 ml 1X PBS solution, and homogenates were shaken for 5 min using a vortex. Swab specimens from animals (feces and saliva swabs), and suspected BU patients (lesions) were treated by adding 2 ml 1X PBS solution into the collection tube followed by repetitive 3min vortexing steps. The content of each collection tube was aseptically transferred into a 2 ml sterile Eppendorf tube, and then centrifuged at 14,000 rpm for 10 min. This procedure was repeated several times until the collection tube was empty. The supernatant was discarded, and the pellet was resuspended in 1X PBS solution, and used for the extraction of genomic DNA. Whatman filter papers containing water filtrands were treated as solid materials. Overall, 250 l of each homogenate was used for DNA extraction. Total genomic DNA (gDNA) was extracted using the QIAGEN DNeasy Blood and Tissue Kit, according to the manufacturers instructions (QIAGEN). Before DNA extraction, MU-free fecal materials from domesticated *Capra aegagrus hircus* (a female goat) were spiked with MU Agy99 pure culture strain and tested for MU-extraction protocol and validation. Contaminations and controls were managed as described previously [17, 37].

#### Quantitative PCR analysis

MU molecular DNA-diagnosis targets (IS*2404*, IS*2606*, and KR-B) were screened from genomic DNA-extracts using the TaqMan qPCR protocol described by Fyfe et al. [38]. Positivity for MU and the cycle threshold cut-offs were defined using the Cts-method (Cts [IS_*2606*_-IS_*2404*_]) as established previously [37, 38]. Multilocus VNTR typing and sequence analysis were also performed as additional confirmatory tests for MU.

#### Genotyping-VNTR profiling

Multilocus VNTR typing of four loci (MIRU1, Locus 6, Locus 19, and ST1) was performed on MU-positive samples to confirm the presence of MU using conditions and primers previously described [17, 30]. Amplicons band sizes and repeat numbers of all loci were estimated according to reference MU-genotyping protocols [39,41]. VNTR genotypes or profiles were defined according to the copy/repeat numbers of each amplified locus and were in the order of MIRU1, Locus 6, ST1, and Locus 19.

#### Sequence analysis

Successfully genotyped amplicons of MIRU1, Locus 6, ST1, and Locus 19 were confirmed with Sanger sequencing as described previously [37]. Briefly, agarose gel fragments containing DNA bands of each locus were purified using the QIAquick Gel Extraction Kit (QIAGEN). Purified amplicons were then subjected to BigDye Terminator v3.1 Cycle Sequencing conditions (Applied Biosystems) by PCR using both forward and reverse primers of each locus. Sequence products were purified using the Dye Terminator Removal Kit (Thermo Fisher Scientific) and cleaned sequences were analyzed in ABI 3500xL Genetic Analyzer using the ABI9500 sequencing program (Applied Biosystems). Sequence trace files were edited in MEGA 7.0 software [42], and consensus sequences generated in FASTA format. Reference sequences of MIRU1 orthologs were retrieved from GenBank.

## Results

### MU-distribution in sampled water bodies and related MU genotypes

Overall, 26 water filtrands sampled from each water body were tested for MU in all investigated sites. The IS*2404* DNA was found in 18 (69.23%) water samples; 10(55.55%) and 6 (33.33%) of these samples were also positive respectively for IS*2606* and KR-B (MU plasmid marker) elements. The difference between Ct-values of IS*2026* and IS*2404* (Cts) was determined for each sample to confirm the presence of MU or other mycolactone-producing mycobacteria (MPMs). Using this analysis, MU was detected in 6 (23.07%) water specimens, including two samples from the Nlong river (W6AC) and a puddle (W2AC) at Akonolinga Centre; one sample (W5ED) collected from a puddle at Edjom; and three specimens originating from the streams (djaa: W2YE; moadjaba: W7YE; nleng: W5YE) at Yeme-Yeme (Table 1). The MU-positive puddles were collected in the vicinity of the BU treatment unit at Akonolinga district hospital and the residence of a local traditional healer specialized in the treatment of chronic wounds at Edjom. The distribution of MU within all sampled water bodies is provided as supporting information to this paper (Additional file 2).

**Table 1:** Distribution of environmental and human samples tested and percentage positive for MU

Samples	Number MU-positive (IS*2404*+IS*2606*+KR-B)/total sampled (%)	MU-genotypes
**Environmental samples**		
Water filtrands	6/26(23%)	D, W
Biofilms	3/22(14%)	D, W, E
Detritus	1/10(10%)	ua
Total	10/58(17%)	/
**Animal samples**		
Feces materials	0/456(0%)	na
Saliva swabs	0/397(0%)	na
Total	0/853(0%)	/
**Clinical samples**		
Human lesion swabs	8/11(73%)	C, D

*MU M*. *ulcerans*, *ua* unasigned, *na* non-applicable

VNTR analysis of the six MU-positive water specimens revealed two MU genotypes D (1,1,2,2) and W (1,1,2,1). The genotype D was found in W6AC collected from the Nlong river at Akonolinga Centre, and W7YE collected from the Moadjaba river at Yeme-Yeme; while genotype W was found in W2YE collected from the Djaa river at Yeme-Yeme. Three unassigned (UA) genotypes characterized by the deletion or non-amplification of MIRU1, Locus 6 and Locus 19 were found in W2AC (UA: 1,0,2,0), W5YE (UA: 1,1,1,0), and W5ED (UA: 0,0,4,0) (Table 2). Indeed, the PCR amplification of VNTR 19 was unsuccessful for W2AC, W5YE, and W5ED; PCR amplifications of Locus 6 was unsuccessful for W2AC and W5ED; and PCR amplification of MIRU 1 was unsuccessful for W5ED. Locus 6 and Locus 19 constituted the main determinants for a genotype in water samples. Random selection and analysis were performed among MU-negative samples, and none carried the MU specific genotypes (Table 2).

**Table 2:** VNTR profile of MU isolates detected in various environmental samples

Localities	Type of samples	Test ID	MIRU-VNTR allelic profile				MU-genotypes
			MIRU-1	Locus-6	ST-1	Locus-19	
Akonolinga Centre	Water body	W6AC	1	1	2	2	D
	Water body	W2AC	1	0	2	0	ua
Yeme-Yeme	Water body	W2YE	1	1	2	1	W
	Water body	W5YE	1	1	1	0	ua
	Water body	W7YE	1	1	2	2	D
	Biofilm	B6YE	1	1	2	2	D
Edjom	Water body	W5ED	0	0	4	0	UA
	Biofilm	B2ED	1	2	1	2	E
Nyeck	Biofilm	B1NY	1	1	2	1	W
Endom	Detritus	D2EN	1	0	1	2	ua

*MU M*. *ulcerans*, *ua* unassigned, 0 no amplification; PCR amplification of VNTR-19 was unsuccessful for W2AC and W5ED; PCR amplification of Locus 6 was unsuccessful for W2AC and D2EN; PCR amplification of MIRU-1 was unsuccessful for W5ED; Locus-6 and Locus-19 were the main determinants for a genotype; Genotype E for B2ED corresponds to *M*. *marinum*

### MU-distribution in plant biofilms and related MU genotypes

A total of 22 plant materials were tested for MU, and 9/22 (40.41%) were IS*2404*-positive. The increased prevalence of IS*2404* in biofilms may be consistent with the suggestion that biofilms play a role in offering a niche environment to MU. In addition to IS*2404*, three biofilm samples (B6YE, B2ED, B1NY) were also positive for IS*2606* and KR-B (Table 1). The MU-positive biofilm samples were collected from the Moadjaba river at Yeme-Yeme, Mezosso river at Edjom, and Koro river at Nyeck. Overall, all MU-positive biofilms were successfully typed and three assigned genotypes were found: D (1,1,2,2), E (1,2,1,2), and W (1,1,2,1). The MU genotype D (1,1,2,2) was found in B6YE collected from the Moadjaba river at Yeme-Yeme, the genotype E (1,2,1,2) was found in B2ED collected from the Mezosso river at Edjom, and the genotype W (1,1,2,1) was found in B1NY collected from the Koro river at Nyeck (Table 2). D (1,1,2,2) and W (1,1,2,1) are two well-characterized MU genotypes, while genotype E (1,2,1,2) has been described as an *M*. *marinum* genotype. Although PCR analysis [(Ct_I*S2606*_-Ct_*IS2404*_) = 2.81 cycles] revealed the presence of MU in sample B2ED, the VNTR profile for this sample were consistent with genotype E which is attributed to *M*. *marinum*. The likely concurrent presence of this genotype in a MU-positive sample may suggest a similar ecological niche for MU and its ancestor, *M*. *marinum*. Further investigations by sequence analysis revealed that this sample belongs to *M*. *marinum* and not MU. These data highlight VNTR's utility as a powerful tool to discriminate mycobacteria species and as an MU-confirmatory method.

### MU distribution in detritus samples

Ten detritus samples were tested for MU, 5/10 (50%) were IS*2404*-positive, and only 1/10 (10%) sample from Endom (D2EN) was positive for MU after detection of the three targets (IS*2404*+IS*2606*+KR-B) (Cts = 2.78 cycles) (Table 1). VNTR Locus 6 was not amplified for this sample, leading to an unassigned genotype UA (1,0,1,2) (Table 2).

### Investigation of MU presence in domestic and wild animal feces in the Nyong valley

A total of 416 fecal samples were tested for the presence of MU. These include 216 fecal materials from the environment and 200 fecal swabs taken from the anus of wild animals (3 porcupine, 2 hedgehog, 8 rats, and 17 mice) and domesticated animals (mainly avian, ovine, canine, and rodents), which share the human microenvironment. Overall, 22 (5.30%) samples were positive for IS*2404* element which occurred more frequently in the fecal matters sampled from the ground (6.9%, 15/216) than anal swabs (3.5%, 7/200). However, no significant difference was found in the distribution of IS*2404* element between soil-collected feces specimens and fecal swabs. Random selection and analysis of these samples (10% of total samples) did not reveal any MU specific genotypes. The negativity of the samples to MU might suggests the presence of other mycolactone producing mycobacteria (MPMs) like *Mycobacterium liflandii* and *Mycobacterium pseudoshotsii*, that also contain the IS*2404* and IS*2606* molecular targets.

### Investigation of MU presence in domestic animal saliva in the Nyong valley

Similar to fecal samples, MU-DNA was not detected in the saliva of animals (Table 1). Out of the 401 saliva swabs screened, IS*2404* element was detected in 27 (6.7%) of these samples, 5.7% were positive for IS*2606*, and none was positive for KR-B. The Cts-values for IS*2404+*IS*2606*-positive samples were not consistent with the identification of MU, indicating that MU was not found in the saliva of domesticated animals in the Nyong valley in Cameroon.

### MU distribution and related MU genotypes in clinical samples from suspected BU patients

Active case search was performed within the study communities during field investigations and environmental samplings. Eleven suspected BU-patients were tested and MU (IS*2404*+IS*2606*+KR-B) was detected in the lesions of eight of the patients (Table 1). The identified BU patients had category I (37%) and category II (63%) ulcerative lesions. VNTR characterization revealed two MU genotypes found in humans: the well-known genotype C (3,1,2,2) and the genotype D (1,1,2,2), initially found in environmental samples (water bodies and biofilms). The genotype C was found in the lesions of 4/6 (66.67%) patients, while the genotype D was found in 2/6 (33.33%) patients. Genotype C (3,1,2,2) has been previously described as the unique MU genotype for clinical samples in Akonolinga. Genotype D (1,1,2,2) is therefore reported for the first time in human patients in this BU endemic locality. PCR amplification of VNTR-19 was unsuccessful for two lesion samples (H1YE and H1NY). No diversity was found in Locus-6 and ST-1, MIRU 1, and Locus 19 indexed diversity and constituted the main determinants for MU genotype (Table 3).

**Table 3:** VNTR profile of MU isolates detected in human lesions

Localities	Type of samples	Test ID	MIRU-VNTR allelic profile				MU-genotypes
			MIRU-1	Locus-6	ST-1	Locus-19	
Akonolinga Centre	Lesion swab	H1AC	3	1	2	2	C
	Lesion swab	H2AC	3	1	2	2	C
Yeme-Yeme	Lesion swab	H1YE	1	1	2	0	ua
	Lesion swab	H3YE	3	1	2	2	C
Edjom	Lesion swab	H2ED	1	1	2	2	D
Nyeck	Lesion swab	H1NY	0	1	2	0	ua
	Lesion swab	H2NY	3	1	2	2	C
Nkolessong	Lesion swab	H1NK	1	1	2	2	D

*MU M*. *ulcerans*, *ua* unassigned, 0 no amplification; PCR amplification of Locus-19 was unsuccessful for H1YE and H1NY; PCR amplification of MIRU-1 was unsuccessful for H1NY; MIRU-1 and Locus-19 were the main determinants for a genotype

### Distribution of MU genotypes in the communities

**Fig. 2:**
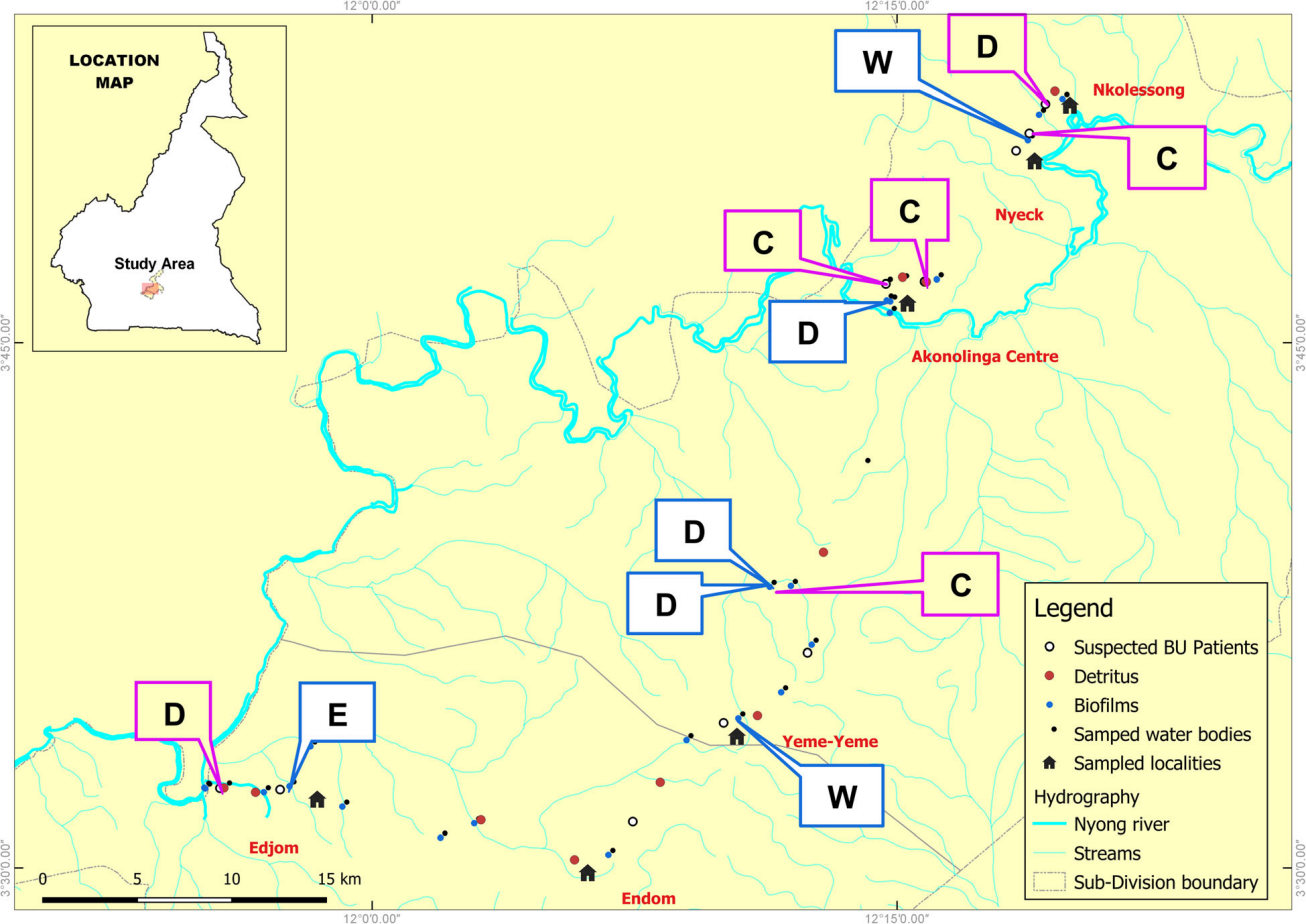
Community-based geographical distribution of *M. ulcerans* genotypes from the environment and humans. Blue square callouts contain genotypes found only in the environment (water bodies and biofilms); Pink square callouts have genotypes found only in human beings; Genotype E corresponds to *M*. *marinum*; Genotype D overlaps between the environment and human populations; blue lines represent water bodies (streams and rivers); Sampled water bodies, biofilms, detritus, and suspected BU patients are represented by colored dots as defined in the legend

The environmental and clinical MU genotypes were overlapped within the study communities (Fig. 2). The data revealed various MU genotypes in the environment and human beings. Two MU genotypes (D, W) were found in the environment, and two other genotypes (C, D) were found in human patients. The micro geo-distribution of MU genotypes within the communities revealed that genotype D was found both in the environment and human patients in Akonolinga health district. On the otherhand, genotype W was detected only in environmental samples in two localities (Yeme-Yeme, Nyeck). Genotype C was specific to human patients (Fig. 2). Overall, from the three MU genotypes found, water bodies harbored 2/3 genotypes (D, W), biofilms had 2/3 (D, W), and BU patients had 2/3 (C, D). All three MU genotypes were simultaneously present in one locality (Yeme-Yeme), while one or two genotypes were found in other localities. No obvious focal grouping of MU genotypes was observed at the community scale. The distribution of MU genotypes within the communities and sampled elements is provided as supplementing material in Additional file 2.

### Sequence analysis of MU genotypes in environmental samples and clinical wounds

**Fig. 3:**
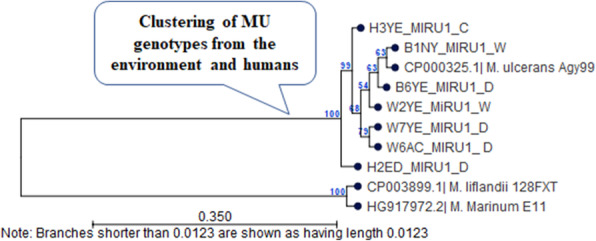
Comparison of MU VNTR profiles between the environment and humans. The relatedness between the genotypes was inferred using the UPGMA method in MEGA 7. Bootstrapping values for 1000 replicates are shown in percentage next to the branches. MIRU1 reference orthologs for *M*. *marinum* (*the ancestor of MU*), *M*. *liflandii* (another MPM), and *M*. *ulcerans* Agy99 (MU reference strain) were retrieved from GenBank with accession numbers given in the tree. MU: *M*. *ulcerans*; Sample test IDs and MU genotypes C, D, W are shown for environmental samples (B1NY, B6YE, W2YE, W7YE, W6AC) and BU lesions (H2ED, H3YE). The figure reveals clustering of MU genotypes from the environment and humans

MU genotypes were found in five and six samples respectively from the environment and humans. Sequence analysis further confirmed the presence of MU in these samples (with >99% sequence identity with the reference sequence of MU-agy99 isolate). Among the VNTR loci investigated, MIRU1 constituted the important polymorphic makers that allow to discriminate environmental and human genotypes of MU. Comparison of the VNTR profiles revealed clustering of environmental and human genotypes (Fig. 3). MU genotypes D and W were close to the outgroup constituted by *M*. *liflandii* and *M*. *marinum* (environmental isolates). Sequence comparisons revealed that the new human genotype D is similar (<5 SNPs) to genotype D found in the environment. Genotypes C and W are more divergent (Fig. 3).

## Discussion

Whole-genome sequence analysis of MU isolates from patients living in endemic villages has so far been the approach that has provided significant insights into how MU genotypes are distributed at the local level and how this pathogen could be transmitted to humans [26,29]. The absence of MU isolates recovered in culture from the environment continues to hinder our ability to draw definitive conclusions on the mode of transmission of BU and other fine epidemiologic details. In this study, we employed VNTR typing to describe the distribution of both environmental and human MU genotypes in a local community setting and to attempt to determine a link between environmental sources of MU with human infection.

A recent study undertaken to determine the distribution pattern of MU employed the high discriminating power of single-nucleotide polymorphism (SNPs) analysis to investigate isolates of patients living in close communities along a river course in the Densu river basin in Ghana [43]. MU haplotypes were identified to form focal clusters within a given local community with little or no mixing of genotypes in the communities. However, one particular haplotype named the founder haplotype was the only type that was widespread across the communities. Similarly, whole-genome analysis of MU isolates of patients living in the Nyong valley and the Map river basin in Cameroon identified local clonal complexes indicating limited distribution of MU genotypes in the localities [28]. The focal distribution pattern was thought to be a result of accumulation of SNPs in local MU populations. A departure from this apparent focal distribution of MU genotypes was revealed for the first time in the BU endemic communities of the Asante Akim north district in Ghana where multiple MU genotypes were present and randomly distributed in communities within a 525 m radius [27]. This pattern of distribution has been attributed to a number of possibilities including the introduction of different genotypes from distant endemic foci into the communities, or dispersal of the genotypes by vectors that move among the communities [27].

The VNTR genotypes C, D, W identified in this study have also been described in previous investigations [12, 30] that also found limited number of genotypes for both patient isolates and environmental samples. MU genotypes D and W were found in environmental samples, and two genotypes C and D were found in clinical samples from BU patients. Genotype D was the primary environmental genotype and was found in almost all of the communities except Endom and Nyeck, and it was also found for the first time in human lesions in the investigated communities. Sequence analysis revealed identical nucleotide sequences of the VNTR loci of the D genotypes (from both the environmental and human sources). These data provide evidence that the genotype D of MU detected in the environmental samples is the same as the genotype which causes disease in humans in this area. Previous studies have detected identical VNTR genotypes in environmental samples (feces, water, biofilms, and mosquitoes), and in human isolates originating from the same endemic area [1, 12, 15, 30]. Genotype W constituted a typical environmental genotype that has also been found in MU-contaminated environmental samples in Ghana [12]. Genotype C in contrast is a well-characterized human genotype of MU circulating in West and Central Africa including Cameroon, Benin, Ivory Coast, and Ghana [12, 17, 30, 31]. Six unassigned genotypes identified were confirmed to be MU by sequence analysis of VNTR loci and the Ct-method [Cts(Ct_IS*2606*_-Ct_IS*2404*_)]. Our findings suggest that MU may be circulating in the six communities of the Akonolinga district with multiple genotypes present in any one local community.

The tributaries of the Nyong river within the communities may carry MU from one community to another, leading to the random distribution of genotypes in the endemic communities. Previous studies have indicated the possibility of human contamination by MU from slow-moving watercourses (e.g., ponds) or other environmental elements like possum feces [1, 12]. Other factors that have been suggested to account for the spread of MU genotypes includes movements of people in the endemic communities and the possible involvement of a vector [26, 27].

MU was not detected in fecal matter and saliva of animals in the study communities. While this observation is consistent with a previous investigation from Ghana [44], it is a marked departure from other studies that found MU in fecal matters shed in the environment by animals and humans [22, 32, 33]. Nevertheless, the application of molecular approaches appears to provide us with some useful insights into how MU may be spreading in endemic communities.

## Conclusion

This study identified different MU VNTR genotypes that were randomly distributed in local endemic communities in a river valley basin in Cameroon. Although these observations may provide useful insights into BU microepidemiology, recovering MU in culture from the contaminated environmental samples remain paramount in our understanding of the mode of transmission of MU and other details of BU epidemiology. MU was not detected in animal fecal materials and saliva excretions screened within the BU endemic foci, suggesting that domestic animals are unlikely to be major sources of MU in the Nyong Valley in Cameroon.

## Supplementary Information

**Additional file 1.** Distribution of sampled water bodies within the study sites in Akonolinga health district.

**Additional file 2.** Distribution of MU-positivity per sampled locality and sample type.

## Data Availability

All data is held in the manuscript and Supporting Information files.

## Abbreviations

BU: Buruli ulcer disease
MU: *Mycobacterium ulcerans*
VNTR: Variable number of tandem repeats
