# Supplementary material for: Community-based geographical distribution of Mycobacterium ulcerans VNTR-genotypes from the environment and humans in the Nyong valley, Cameroon
Source: Trop Med Health. 2021 May 21;49:41. doi: 10.1186/s41182-021-00330-2 (PMC8139057; doi:10.1186/s41182-021-00330-2)
Supplement: Supplementary file 1 — Additional file 1. Distribution of sampled water bodies within the study sites in Akonolinga health district. [file 41182_2021_330_MOESM1_ESM.docx]

**Additional file 1** : Distribution of sampled water bodies within the study sites in Akonolinga health district.

| **Localities** | **Nature of water body** | **Local name** | **TEST ID** | **MU-Distribution** | **Mu-genotype** |
| --- | --- | --- | --- | --- | --- |
| Akonolinga Centre | stream | DAMNYALA | W1AC | Neg |  |
|  | Puddle | / | W2AC | Pos | UA |
|  | Swamp | / | W3AC | Neg |  |
|  | River | NLONG 1 | W4AC | Neg |  |
|  | River | NLONG 2 | W5AC | Neg |  |
|  | River | NLONG 3 | W6AC | Pos | D |
| Yeme-Yeme | Stream | MBEDE | W1YE | Neg |  |
|  | Stream | DJA'A | W2YE | Pos | W |
|  | Stream | VINGUI | W3YE | Neg |  |
|  | Stream | WOMBO | W4YE | Neg |  |
|  | Stream | NLENG | W5YE | Pos | UA |
|  | Stream | MOADJABA | W7YE | Pos | D |
|  | Stream | MVABA | W6YE | Neg |  |
| Endom | Stream | NGA'A WULU 1 | W1EN | Neg |  |
|  | Stream | NGA'A WULU 2 | W2EN | Neg |  |
| Edjom | Stream | NGOUNOU | W1ED | Neg |  |
|  | Stream | MEZOSSO | W2ED | Neg |  |
|  | River | NLONG 1 | W3ED | Neg |  |
|  | River | NLONG 2 | W4ED | Neg |  |
|  | Puddle | / | W5ED | Pos | UA |
|  | Stream | MEVOULOU | W6ED | Neg |  |
|  | Stream | KOMENA | W7ED | Neg |  |
|  | Stream | ETOUM-ZOUMOU | W8ED | Neg |  |
| Nyeck | Stream | KORO | W1NY | Neg |  |
|  | Stream | NKENDE | W2NY | Neg |  |
| Nkolessong | Stream | NLONG | W1NK | Neg |  |

MU, *M. ulcerans*; UA, Unassigned; Pos, Positive; Neg, Negative.
