# Supplementary material for: Community-based geographical distribution of Mycobacterium ulcerans VNTR-genotypes from the environment and humans in the Nyong valley, Cameroon
Source: Trop Med Health. 2021 May 21;49:41. doi: 10.1186/s41182-021-00330-2 (PMC8139057; doi:10.1186/s41182-021-00330-2)
Supplement: Supplementary file 2 — Additional file 2. Distribution of MU-positivity per sampled locality and sample type. [file 41182_2021_330_MOESM2_ESM.docx]

**Additional file 2**: Distribution of MU-positivity per sampled locality and sample type.

| **Localities** | **Type of sample** | **total sampled** | **MU-positivity** | **MU-Percentage (%)** | **MU-genotype** |
| --- | --- | --- | --- | --- | --- |
| **Akonolinga Centre** | Water body | 6 | 2 | 33.33% | D |
|  | Biofilm | 4 | 0 | 0.00% | ND |
|  | Detritus | 2 | 0 | 0.00% | ND |
|  | Saliva swabs | 50 | 0 | 0.00% | ND |
|  | Feces materials | 63 | 0 | 0.00% | ND |
|  | Human lesion swabs | 2 | 2 | 100.00% | C |
| **Yeme-Yeme** | Water body | 7 | 3 | 42.86% | D, W |
|  | Biofilm | 6 | 1 | 16.67% | D |
|  | Detritus | 2 | 0 | 0.00% | ND |
|  | Saliva swabs | 66 | 0 | 0.00% | ND |
|  | Feces materials | 57 | 0 | 0.00% | ND |
|  | Human lesion swabs | 3 | 2 | 66.67% | C |
| **Endom** | Water body | 2 | 0 | 0.00% | ND |
|  | Biofilm | 2 | 0 | 0.00% | ND |
|  | Detritus | 3 | 1 | 33.33% | UA |
|  | Saliva swabs | 55 | 0 | 0.00% | ND |
|  | Feces materials | 49 |  | 0.00% | ND |
|  | Human lesion swabs | 1 | 0 | 0.00% | ND |
| **Edjom** | Water body | 8 | 1 | 12.50% | UA |
|  | Biofilm | 7 | 1 | 14.29% | E |
|  | Detritus | 2 | 0 | 0.00% | ND |
|  | Saliva swabs | 118 | 0 | 0.00% | ND |
|  | Feces materials | 146 | 0 | 0.00% | ND |
|  | Human lesion swabs | 2 | 1 | 50.00% | D |
| **Nyeck** | Water body | 2 | 0 | 0.00% | ND |
|  | Biofilm | 2 | 1 | 50.00% | W |
|  | Saliva swabs | 75 | 0 | 0.00% | ND |
|  | Feces materials | 90 | 0 | 0.00% | ND |
|  | Human lesion swabs | 2 | 2 | 100.00% | C |
| **Nkolessong** | Water body | 1 | 0 | 0.00% | ND |
|  | Biofilm | 1 | 0 | 0.00% | ND |
|  | Detritus | 1 | 0 | 0.00% | ND |
|  | Saliva swabs | 33 | 0 | 0.00% | ND |
|  | Feces materials | 51 | 0 | 0.00% | ND |
|  | Human lesion swabs | 1 | 1 | 100.00% | D |

MU, *M. ulcerans*; UA, Unassigned; ND, non-determined.
